# Chemopreventive Potential of 2,3,5,4′-Tetrahydroxystilbene-2-O-*β*-D-glucoside on the Formation of Aberrant Crypt Foci in Azoxymethane-Induced Colorectal Cancer in Rats

**DOI:** 10.1155/2017/3634915

**Published:** 2017-11-07

**Authors:** Chien-Liang Lin, Jiiang-Huei Jeng, Chih-Chung Wu, Shu-Ling Hsieh, Guan-Cheng Huang, Wan Leung, Chining-Ting Lee, Chung-Yi Chen, Chien-Hsing Lee

**Affiliations:** ^1^Department of Medical Education and Research and Department of Pharmacy, Yuan's General Hospital, Kaohsiung, Taiwan; ^2^Institute of Clinical Dentistry and Department of Dentistry, National Taiwan University Hospital and National Taiwan University Medical College, Taipei, Taiwan; ^3^Department of Nutrition and Health Sciences, Chang Jung Christian University, Tainan, Taiwan; ^4^Department of Seafood Science, National Kaohsiung Marine University, Kaohsiung, Taiwan; ^5^Department of Health-Business Administration, School of Nursing, Fooyin University, Kaohsiung, Taiwan; ^6^Department of Radiology and Nuclear Medicine, Yuan's General Hospital, Kaohsiung, Taiwan; ^7^Department of Nutrition and Health Science, School of Medical and Health Sciences, Fooyin University, Kaohsiung, Taiwan; ^8^Department of Pharmacology, Graduate Institute of Medicine, College of Medicine, Kaohsiung Medical University, Kaohsiung, Taiwan

## Abstract

2,3,5,4′-Tetrahydroxystilbene-2-*O*-*β*-D-glucoside (THSG) has been shown to have antioxidative and anti-inflammatory effects. Oxidative and inflammatory reactions are related to the development of colorectal carcinoma (CRC). In the present study, we characterized the preventive activities of THSG on colon carcinogenesis using the azoxymethane- (AOM-) mediated rat colon carcinogenesis model. F344 male rats were randomly divided into 5 groups (untreated and AOM model rats treated with or without THSG at 30, 150, or 250 mg/kg) after which the numbers of aberrant crypt foci (ACF) were assessed in the colon tissues of all rats. The expressions of nuclear factor-*κ*B (NF-*κ*B), cyclooxygenase-2 (COX-2), matrix metalloproteinase proteins (MMPs), and carcinoembryonic antigen (CEA) were measured as effective early predictors of CRC using western blot analysis. Treatment with THSG (150 or 250 mg/kg) induced a 50% reduction in total colonic ACF formation (*P* < 0.05). Furthermore, our results revealed a downregulation of CEA and NF-*κ*B protein levels in the reduced number of ACF elicited by treatment with THSG, whereas levels of COX-2 and MMPs proteins were not changed. Collectively, THSG may be a promising natural lead compound or drug candidate for treating early phases of CRC.

## 1. Introduction

Colorectal carcinoma (CRC) is one of the most common tumors in the world and is undoubtedly an important health problem among the Asian population [1]. CRC is the leading cause of cancer deaths in Taiwan and the death rate was 23.9 per 100,000 in 2014 [2]. CRC is widely recognized as curable if caught and treated in the initial stage compared with numerous other cancers [3]. To reduce the mortality due to CRC, the search for biomarkers useful for the early detection of CRC is of the utmost importance.

Tumor markers, such as matrix metalloproteinase proteins (MMPs) and carcinoembryonic antigen (CEA), reflect tumor biology and have the potential to solve a variety of clinical needs. In particular, MMP-2 and MMP-9 have been suggested to be associated with prognosis in CRC patients [4–6]. Several studies have shown that the aberrant activation of NF-*κ*B is frequently found in both experimental and human CRC [7, 8], demonstrating that decreased NF-*κ*B levels could be protective against the growth of CRC. In addition, levels of CEA have been shown to increase about 90% in advanced CRC and to reflect the characteristics of malignant CRC [9, 10]. In other words, high levels of CEA are causally correlated with the progression of CRC [11], implying that reducing the expression of CEA can be used for CRC treatment strategies.

In addition, the earliest distinguishable neoplastic lesions in CRC models are aberrant crypt foci (ACF) [12]. Monitoring ACF is therefore utilized to evaluate anticarcinogenic effects against CRC [13]. Moreover, the initiation phase of CRC has been frequently induced by the carcinogen azoxymethane (AOM), and thus ACF formation induced by AOM can be applied to experimental models [14].

**Figure 1:**
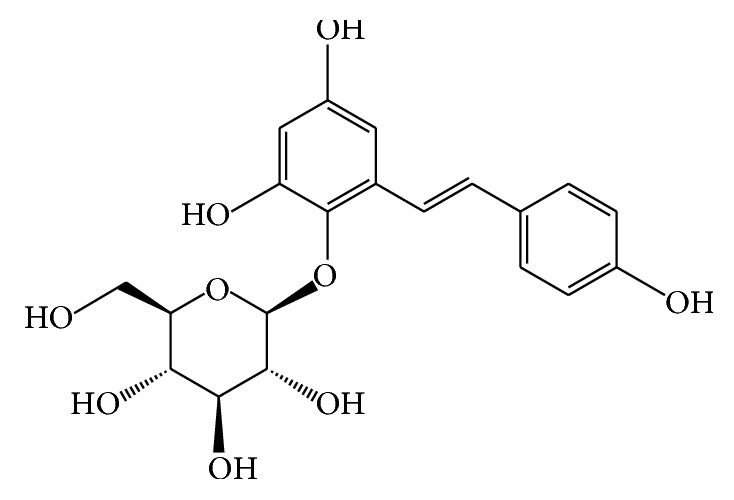
Chemical structure of THSG (2,3,5,4′-tetrahydroxystilbene-2-*O*-*β*-D-glucoside, C_20_H_22_O_9_, molecular weight 406).

2,3,5,4′-Tetrahydroxystilbene-2-O-*β*-D-glucoside (THSG; Figure 1) is an active component derived from the medical herb* Polygonum multiflorum* Thunb (He-Shou-Wu). THSG possesses a great number of pharmacological properties including antilipemia and cardioprotective effects, as well as free radical-scavenging, antioxidant, and anti-inflammation effects [15–17]. Traditional medicinal plants with antioxidant and anti-inflammatory activities have been used for the treatment of AOM-induced ACF [18–20]. However, the potential preventive effects of THSG for CRC* in vivo* have not been studied. Here we investigated whether THSG can exhibit a chemopreventive effect on the AOM-induced CRC model.

## 2. Materials and Methods

### 2.1. Chemicals and Reagents

THSG (HPLC purity 95%) was purchased from the Phytochemistry Laboratory, Department of Pharmacology, Tongji Medical College, Huazhong University of Science & Technology (China). Antibodies to COX-2, MMP-2, MMP-9, NF-*κ*B, and CEA were purchased from R&D Systems (Minneapolis, MN, USA). The monoclonal antibody to *β*-actin was obtained from Sigma (St. Louis, MO, USA).

### 2.2. Experimental Animals

Thirty 4-week-old male F144 rats were obtained from the National Laboratory Animal Center (NLAC) in Taiwan. This study was carried out in accordance with the approval of the Ethics Committee for Animal Experimentation, Chang Jung Christian University, Taiwan [Ethic number CJCU-100-009] and was in compliance with the “Guide for the Care and Use of Laboratory Animals.”

### 2.3. Experimental Protocol

Rats were randomly divided into five groups of six rats each: normal, AOM model (obtained by subcutaneous injections of 15 mg/kg AOM once per week for 3 consecutive weeks), and rats treated with or without THSG (30, 150, or 250 mg/kg). In the THSG-treated groups, the rats were treated orally with THSG (30, 150, and 250 mg/kg) for 6 weeks, after the colon carcinogenesis had been induced by AOM. Rats were sacrificed at 13 weeks of age in a CO_2_ chamber and tissues were evaluated for ACF in the colon as well as biochemical analysis to determine functions of the heart, liver, kidney, and spleen. These specimens were fixed in 10% buffered formalin and were embedded in paraffin using an automated tissue processing machine (Leica, Nussloch, Germany). Sections were cut at 5 *μ*m and were then stained with haematoxylin and eosin (H&E) for histological evaluation.

### 2.4. Determination of ACF

Topographical analysis of the colonic mucosa was done according to Bird as routinely performed [21]. After fixation for at least 24 h at 4°C, the sigmoid colons were cut (about 1 cm) and stained with 0.5% methylene blue (in H_2_O) for 15 sec and then were examined for the total number of ACF by light microscopy.

### 2.5. Western Blot Analysis

Tissue samples (sigmoid colons) in lysis buffer (Pierce, USA) were sonicated for 3 sec and centrifuged and aliquots denatured at 70°C for 10 min in SDS sample buffer and separated by SDS-PAGE. The bands were visualized using hydrogen peroxide/diaminobenzidine tetrahydrochloride or an enhanced chemiluminescence detection kit (Amersham Life Science, Buckinghamshire, UK); then, the band densities were quantified using an AlphaImager 2000 imaging system (Alpha Innotech, San Leandro, CA, USA). All antibodies were used for western blots at a 1 : 1000 dilution and were visualized by enhanced chemiluminescence (SuperSignal West Femto Kit, Pierce; Rockford, IL, USA). Densitometric analysis of protein bands was calculated based on *β*-actin as a loading control.

### 2.6. NF-*κ*B p65 DNA Binding Activity Assay

A TranAM™ NF-*κ*B p65 Chemi Transcription Factor Assay Kit was utilized (Active Motif, Carlsbad, CA, USA) to evaluate NF-*κ*B p65 DNA binding activity according to the manufacturer's recommendation [22].

### 2.7. Statistical Analyses

Data were analyzed statistically using SigmaStat 2.03 software (Jandel Scientific, San Rafael, CA, USA). Significant differences were determined by one-way analysis of variance (one-way ANOVA) followed by a paired *t*-test for multiple comparisons. Data are presented as means ± SEM. *P* < 0.05 is considered statistically significant.

## 3. Results

### 3.1. General Observations

No significant difference in body weight gain or diet intake was determined among the control and THSG-treated groups of rats throughout the study (Table 1). Furthermore, necropsy analysis revealed no pathological modifications in gross visual observations of organs including the heart, liver, kidney, and spleen among all groups of rats (Table 2). These results demonstrated that there were no possible adverse side effects or any gross signs of toxicity induced by THSG in AOM-induced ACF in rats.

### 3.2. Effects of THSG in AOM-Induced ACF Number

**Figure 2:**
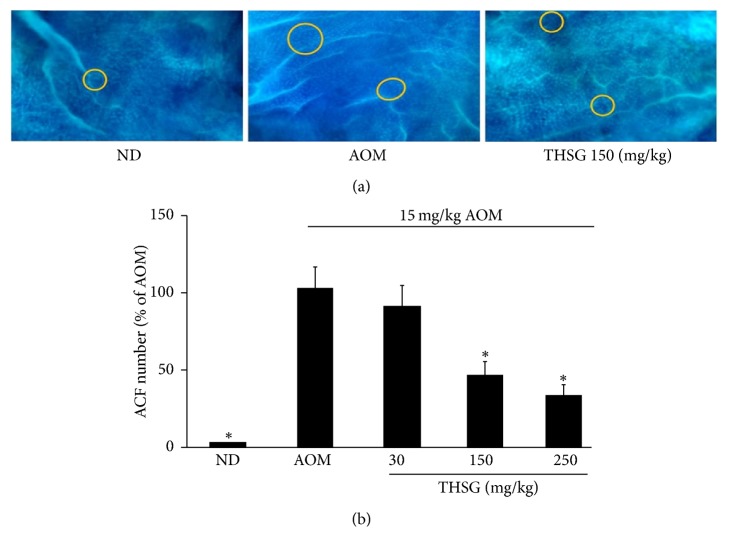
Effects of THSG on AOM-induced ACF number in rat colon. F344 rats were given subcutaneous injection of AOM (15 mg/kg body weight) once a week for 3 weeks after different doses of THSG (30, 150, or 250 mg/kg) for 6 weeks. (a) AOM-induced colorectal ACF stained with methylene blue. (b) Effects of THSG-treatments on ACF formation. Data represent the mean ± SE. ^*∗*^*P* < 0.05. ND represents normal group.

ACF were visualized by staining with methylene blue (Figure 2(a)). The administration of AOM alone induced a total number of ACF around 100%. The inhibitory effects of dietary THSG (150 and 250 mg/kg) on ACF formation in AOM-induced CRC were significantly produced in a concentration-dependent manner compared with the AOM alone (*n* = 6, 50 ± 3.47 versus 105 ± 1.54; 45 ± 1.23 versus 100 ± 1.54, *P* < 0.05, respectively; Figure 2(b) and Table 3). However, the decrease in ACF number at the lowest dose (30 mg/kg) of THSG was not statistically significant. Thus, in the remainder of this study, we performed the experiments using 150 and 250 mg/kg THSG.

Taken together, THSG (150 and 250 mg/kg) resulted in 47 and 54% reduction in the number of ACF, respectively (*P* < 0.05; Table 3), demonstrating that THSG is a promising chemopreventive agent for the chemical carcinogen-induced model of CRC.

### 3.3. Effects of THSG on Tumor Angiogenesis and the Expression of Major Proangiogenic Factors in Colon Tissue from AOM-Induced Rats

**Figure 3:**
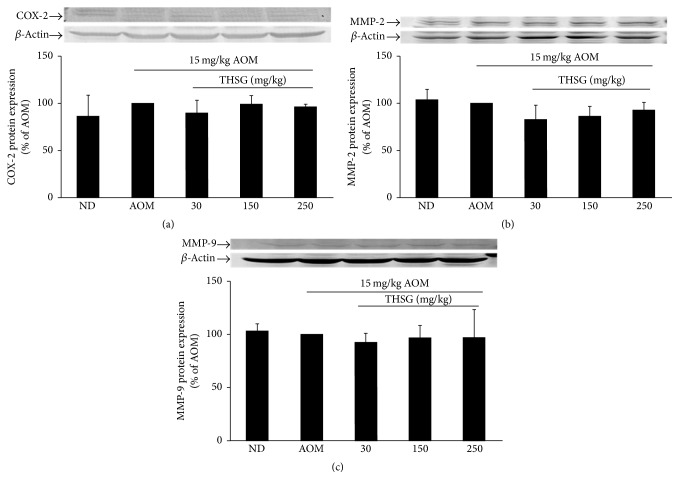
Effects of THSG on tumor angiogenesis and the expression of proangiogenic factors in the colon tissue from AMO-exposed rats. Rat was given THSG with different concentrations (30, 150, or 250 mg/kg) for 6 weeks. The level of COX-2 (a), MMP-2 (b), and MMP-9 (c) protein was determined by western blot analysis with *β*-actin used as the internal control. Data represent the mean ± SE. ND represents normal group.

The formation of new blood vessels contributes to tumor growth and is under the regulation of important proangiogenic factors such as MMP-2 and MMP-9 [23]. Moreover, overexpression of COX-2 has also been shown to be involved in tumor angiogenesis [24]. Therefore, we next examined whether THSG reduces tumor growth by prohibiting its vascularization. The expression levels of COX-2, MMP-2, and MMP-9 in colonic tissues were evaluated by western blotting analysis using specific antibodies. The expression levels of COX-2, MMP-2, and MMP-9 proteins were not changed in AOM-induced ACF treated with THSG at 150 or 250 mg/kg (Figure 3). These results revealed that COX-2, MMP-2, and MMP-9 proteins are not associated with the preventive effect of THSG on AOM-induced ACF.

### 3.4. Effects of THSG on the Nuclear Translocation of NF-*κ*B in Colon Tissues of AOM-Induced Rats

**Figure 4:**
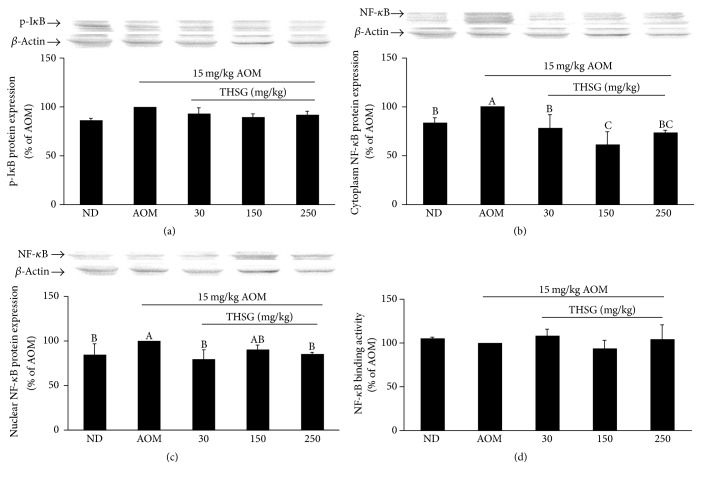
Effects of THSG on nuclear translocation of NF-*κ*B in AOM-induced tissue. Rat was given THSG with different concentrations (30, 150, or 250 mg/kg BW) for 6 weeks. The level of p-I*κ*B protein (a), cytoplasm (b), and nuclear (c) NF-*κ*B protein, and NF-*κ*B binding activity (d), was determined by western blot analysis with *β*-actin used as the internal control. Data represent the mean ± SE. Different alphabet letters (A, B, C) indicate that the NF-*κ*B protein expressions are statistically different from each other (*P* < 0.05). ND represents normal group.

A decrease of NF-*κ*B levels has been shown to protect against the growth of CRC [7, 8]. When bound by the inhibitor protein I*κ*B, NF-*κ*B is retained in the cytoplasm resulting in an inactive state [25]. The induced phosphorylation of I*κ*B results in the upregulation of NF-*κ*B nuclear translocation and the subsequent expression of its target genes. THSG at 150 and 250 mg/kg did not influence the expression level of I*κ*B in AOM-induced ACF formation (Figure 4(a)). In addition, a reduction of NF-*κ*B in the cytoplasm and nucleus was observed (Figures 4(b) and 4(c)), whereas its DNA binding activities were not suppressed by treatment with THSG (Figure 4(d)). These results indicate that THSG suppresses NF-*κ*B activation by inhibiting the translocation of NF-*κ*B.

### 3.5. Effects of THSG on the Tumor Marker CEA in Colon Tissue from AOM-Induced Rats

**Figure 5:**
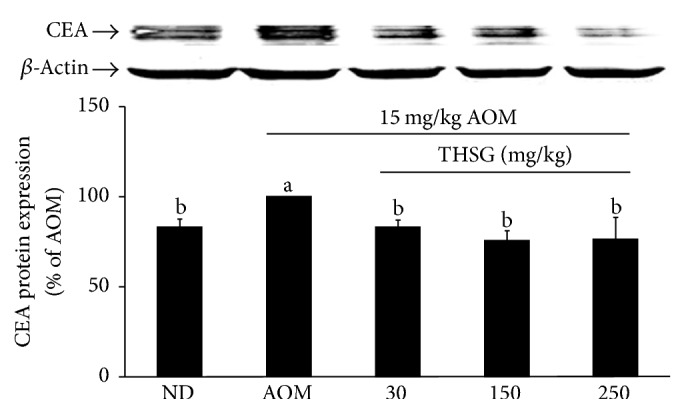
Effects of THSG on CEA protein expression in AOM-induced colonic cancer in rats. F344 rat was given THSG with different concentrations (30, 150, or 250 mg/kg) for 6 weeks. The level of CEA protein was determined by western blot analysis with *β*-actin used as the internal control. Data represent the mean ± SE. Different alphabet letters (a, b) indicate that the CEA expressions are statistically different from each other (*P* < 0.05).

CEA has clinical significance as a classical CRC marker, and it has been used as a prognostic factor for CRC patients and to monitor CRC recurrence [26]. CEA protein was significantly increased in AOM-induced ACF compared with the normal control group. Treatment with THSG at 150 or at 250 mg/kg significantly decreased the levels of CEA (*P* < 0.05) (Figure 5).

## 4. Discussion

In the present study, we characterized the potential biological activity of THSG to prevent colonic inflammation of AOM-treated rats, and its possible inhibitory molecular mechanism on carcinogenesis of the colon was examined. These results provide new insights on the effects of THSG and colon cancer prevention.

The pathogenesis of CRC involves the multistep deregulation of epithelial cells into polyps as colon cancer progresses to a cancerous state. ACF are evaluated as a biomarker for the early-stage diagnosis of CRC [27]. Therefore, reductions of ACF number are used to estimate the effects of potential chemopreventive agents against CRC. In the present study, ACF were induced after AOM injection and occurred early during colorectal tumorigenesis, consistent with previous findings [28, 29]. The present findings show that THSG significantly reduces the total number of ACF induced by AOM compared with the AOM alone group. These observations suggest that THSG can suppress the AOM-stimulated CRC during the initiation stage.

CEA, a typical tumor marker in CRC, has been shown to be associated with recurrent and advanced disease, as well as a poor therapeutic outcome [30]. CEA is distributed in all CRC tissues, even if with distinct ratios of positively stained cells. A clinical report has demonstrated that CRC patients with high expression levels of CEA have a higher risk of relapse and prognosis than those with low expression levels of CEA [31]. CEA belongs to the super-immunoglobulin gene family, which includes genes encoding a number of adhesion proteins, such as intercellular adhesion molecule 1 (ICAM-1) and lymphocyte function-associated antigen 1 [32]. An elevated or rising CEA level has been demonstrated to contribute to reinforcing the presence of the tumor and enhancing its metastatic potential, because the invasion and metastasis of tumors commonly result from alterations in cell adhesion [33, 34]. Meanwhile, our previous studies have shown that THSG abolishes the metastasis of HT29 colon cancer cells [35]. Therefore, regulating the level of expression of CEA in CRC is an attractive strategy to determine the effectiveness of therapy.

Several studies have demonstrated that treatment with resveratrol is a promising strategy to treat colon cancer by the suppression of inflammatory mediators and the reduction of oxidative stress [36–38]. The structure of THSG is similar to resveratrol but also has a polyphenolic group (Figure 1). Phenolic compounds have been shown in many studies to have antitumor, proapoptosis, and antiangiogenesis effects. Further, our results show that THSG suppresses AOM-induced ACF formation by inhibiting the NF-*κ*B pathway. Meanwhile, several studies have proven that CEA levels of CRC patients are significant indicators of their prognosis and postoperative survival rate. In the present study, we found that THSG reduces CEA levels in AOM-induced ACF formation. Taken together, THSG may be a potential candidate for CRC treatment.

In conclusion, this study shows that levels of NF-*κ*B and CEA proteins are significantly elevated in AOM-stimulated ACF compared to the THSG-treated groups. Moreover, the activity of CEA and the translocation of NF-*κ*B are suppressed by the administration of THSG, demonstrating that THSG inhibits the formation of AOM-induced ACF through NF-*κ*B and CEA.

## Figures and Tables

**Table 1:** Effects of THSG on growth characteristics in AOM-induced ACF in rats.

	Food intake (g/d)		Body weight gain (g)	
	3 wks	9 wks	3 wks	9 wks
Normal	14.00 ± 0.56	21.22 ± 0.60	121.3 ± 12.65	256.8 ± 12.65
AOM only	14.78 ± 0.15	21.92 ± 0.52	133.2 ± 19.56	270.7 ± 17.36
30 mg THSG	14.45 ± 0.36	21.69 ± 0.26	125.3 ± 20.38	253.3 ± 23.56
150 mg THSG	14.39 ± 0.26	21.33 ± 0.56	127.3 ± 23.12	250.7 ± 24.08
250 mg THSG	14.26 ± 0.28	21.86 ± 0.24	124.7 ± 22.83	255.2 ± 18.72

F344 rats were treated orally with different doses of THSG (30, 150, or 250 mg/kg) for 6 weeks. The average initial body weights among these groups ranged from 243 to 270 g. Data are expressed as means ± SD (*n* = 6).

**Table 2:** Effects of THSG on organ weight in AOM-induced ACF in rats.

	Heart	Liver	Kidney	Spleen
	% of body weight			
Normal	0.34 ± 0.01	3.27 ± 0.06	0.83 ± 0.01	0.21 ± 0.02
AOM only	0.32 ± 0.01	3.22 ± 0.07	0.83 ± 0.01	0.20 ± 0.01
30 mg/kg THSG	0.32 ± 0.01	3.32 ± 0.04	0.84 ± 0.02	0.22 ± 0.01
150 mg/kg THSG	0.32 ± 0.01	3.35 ± 0.14	0.85 ± 0.02	0.21 ± 0.01
250 mg/kg THSG	0.32 ± 0.01	3.30 ± 0.06	0.85 ± 0.03	0.20 ± 0.02

F344 rats were treated orally with different doses of THSG (30, 150, or 250 mg/kg) for 6 weeks. Data are expressed as means ± SD (*n* = 6).

**Table 3:** Effects of THSG on AOM-induced ACF formation in rats.

Group	ACF number	
	Total	Inhibition (%)
ND (normal)	0	0
AOM 15 mg/kg	105 ± 1.54	0
AOM + THSG (30 mg/kg)	89 ± 9	1.6
AOM + THSG (150 mg/kg)	50 ± 3.47^*∗*^	46.5
AOM + THSG (250 mg/kg)	45 ± 1.23^*∗*^	53.8

F344 rats were treated orally with different doses of THSG (30, 150, or 250 mg/kg) for 6 weeks. Data are expressed as means ± SD (*n* = 6). ^*∗*^*P* < 0.05.
